# A quantitative three-dimensional analysis of posterior facet displacement in Sanders Type II and III intra-articular calcaneal fractures

**DOI:** 10.3389/fsurg.2026.1839210

**Published:** 2026-07-21

**Authors:** Xinzhe Ma, Shibo Zhai, Shuo Zhang, Pengfei Shen, Luqin Di, Wei Chen, Zhiyong Hou, Lijie Ma

**Affiliations:** 1 Department of Orthopaedic Surgery, Hebei Medical University Third Hospital, Shijiazhuang, Hebei, China; 2 Emergency Department, The Second Hospital of Shijiazhuang, Shijiazhuang, Hebei, China; 3 NHC Key Laboratory of Intelligent Orthopaedic Equipment, Shijiazhuang, Hebei, China; 4 Engineering Research Center of Orthopedic Minimally Invasive Intelligent Equipment, Ministry of Education, Shijiazhuang, Hebei, China; 5 Key Laboratory of Biomechanics of Hebei Province, Shijiazhuang, Hebei, China

**Keywords:** calcaneal fractures, displacement characteristics, intra-articular fracture, posterior facet, quantitative analysis, rotation

## Abstract

**Objective:**

To quantify the three-dimensional (3D) displacement characteristics of the posterior facet fragment in Sanders type II and III intra-articular calcaneal fractures (IACFs) caused by falls from height, thereby providing a quantitative anatomical reference for reduction planning.

**Methods:**

We retrospectively analyzed CT data from 71 patients with closed Sanders type II/III IACFs. Mimics and 3-matic software were utilized for 3D reconstruction and simulated reduction. The translational and rotational changes of the fragment's geometric center were measured before and after reduction.

**Results:**

Significant displacement occurred in all directions (*P* < 0.05). Predominant translations were lateral (4.55 ± 3.16 mm) and inferior (3.81 ± 3.13 mm), while posterior translation was minor. Primary rotational deformities included axial plane internal rotation (17.62 ± 14.57°) and sagittal plane anterior tilt (12.29 ± 13.42°). Coronal plane varus (4.61 ± 13.97°) was minimal but exhibited high variability, indicating significant instability.

**Conclusion:**

Posterior facet fragments primarily displace inferolaterally with significant internal rotation (∼17.62°) and anterior tilt. These findings suggest that rotational malalignment, particularly axial-plane internal rotation and sagittal-plane anterior tilt, may be an important but easily overlooked component of posterior facet displacement. The results may provide a quantitative anatomical reference for preoperative planning and intraoperative assessment of reduction, although further biomechanical and clinical validation is required.

## 1 Introduction

The calcaneus, the largest tarsal bone, bears approximately 60% of the body's weight. Calcaneal fractures account for 1%–2% of all fractures, with intra-articular fractures (IACFs) comprising about 75% (1–4). Displaced IACFs are typically high-energy injuries, resulting from forces such as compression, shear, traction, and direct impact (5–9). These forces concentrate on the posterior facet, leading to multi-directional displacement (7). The complex anatomy of the calcaneus, particularly the posterior facet, is critical for maintaining gait stability and motor function. High-energy trauma (e.g., falls from height, traffic accidents) frequently causes IACFs, with approximately 75% affecting the posterior facet (10).

Currently, the clinical assessment of calcaneal fracture displacement primarily relies on plain radiographs and two-dimensional (2D) computed tomography (CT). However, these conventional 2D imaging modalities have several limitations (8). First, the intricate anatomy of the calcaneus, especially the complex three-dimensional structure of the posterior facet, cannot be comprehensively visualized or intuitively assessed in single planes, leading to an incomplete understanding of the displacement. Second, variations in patient positioning during imaging can introduce measurement errors, compromising the objectivity and reproducibility of findings. These constraints preclude a precise assessment of posterior facet fragment displacement, thereby limiting the clinician's decision-making capacity for preoperative planning and intraoperative execution.

In recent years, the rapid advancement of digital medical technology, particularly the application of medical image processing software such as Mimics 21.0 and 3-matic, has opened new avenues for morphological research in orthopedics (11, 12). 3D reconstruction technology enables clinicians and researchers to observe fracture morphology from any angle and perform high-precision spatial measurements. This technology not only enhances fracture assessment accuracy but also provides robust support for preoperative simulation and the development of personalized treatment plans. Currently, applications of 3D reconstruction in calcaneal fracture research have primarily focused on morphological description, preoperative planning, and the design and fabrication of 3D-printed surgical guides. For instance, 3D models allow surgeons to intuitively assess fracture line distribution and the degree of articular surface collapse, and even to create patient-specific surgical guides to improve surgical precision (13–15). However, a systematic, quantitative investigation of the spatial displacement characteristics of the posterior facet fragment remains absent. This data scarcity limits a deeper understanding of displacement patterns and impedes the optimization of clinical treatment strategies.

In the present study, we employed Mimics and 3-matic software for three-dimensional reconstruction and simulated anatomical reduction of Sanders type II and III intra-articular calcaneal fractures caused by falls from height. By measuring the translational and rotational changes of the posterior facet fragment before and after virtual reduction, we aimed to quantitatively characterize its three-dimensional displacement pattern. Based on the anatomy of the subtalar joint and the mechanism of axial loading, we further explored whether the observed imaging findings were consistent with a possible helical displacement pattern. This concept was used only as an explanatory framework and was not intended to establish a definitive biomechanical mechanism.

## 2 Materials and methods

### 2.1 Study population

This retrospective cohort study included imaging data from 71 patients with closed Sanders type II or III calcaneal fractures caused by falls from height, treated between July 2023 and December 2024. The study protocol was approved by the Institutional Review Board (IRB) of Hebei Medical University Third Hospital (IRB No. Ke2024-115-1). All cases were evaluated and included by senior attending orthopedic surgeons.

#### 2.1.1 Inclusion criteria

Inclusion criteria were: (1) age between 18 and 65 years; (2) unilateral, closed calcaneal fracture; (3) definitive fracture involvement of the posterior facet with observable articular collapse or displacement on imaging; and (4) foot and ankle CT scan completed within one week of injury.

#### 2.1.2 Exclusion criteria

Exclusion criteria were: (1) pathological fractures; (2) old fractures (>1 month); (3) concomitant fractures in other locations; and (4) history of previous surgery on the ipsilateral foot or ankle.

A total of 71 eligible patients were included in this study. All fractures were caused by falls from height, conforming to the study's specific inclusion criteria for injury mechanism.

### 2.2 Equipment and software

Mimics 21.0 and 3-Matic 13.0 (Materialise NV, Belgium) were used as the core tools for medical image processing, 3D reconstruction, and data analysis. These software platforms are FDA 510(k) cleared for medical image processing and preoperative planning. Previous validation studies comparing Mimics-generated bone models to physical cadaveric measurements have demonstrated sub-millimeter accuracy (mean absolute deviation 0.03–0.32 mm) and high inter-observer reproducibility (ICC > 0.90) for orthopedic morphometry.

#### 2.2.1 Fracture fragment segmentation and 3D reconstruction

**Figure 1:**
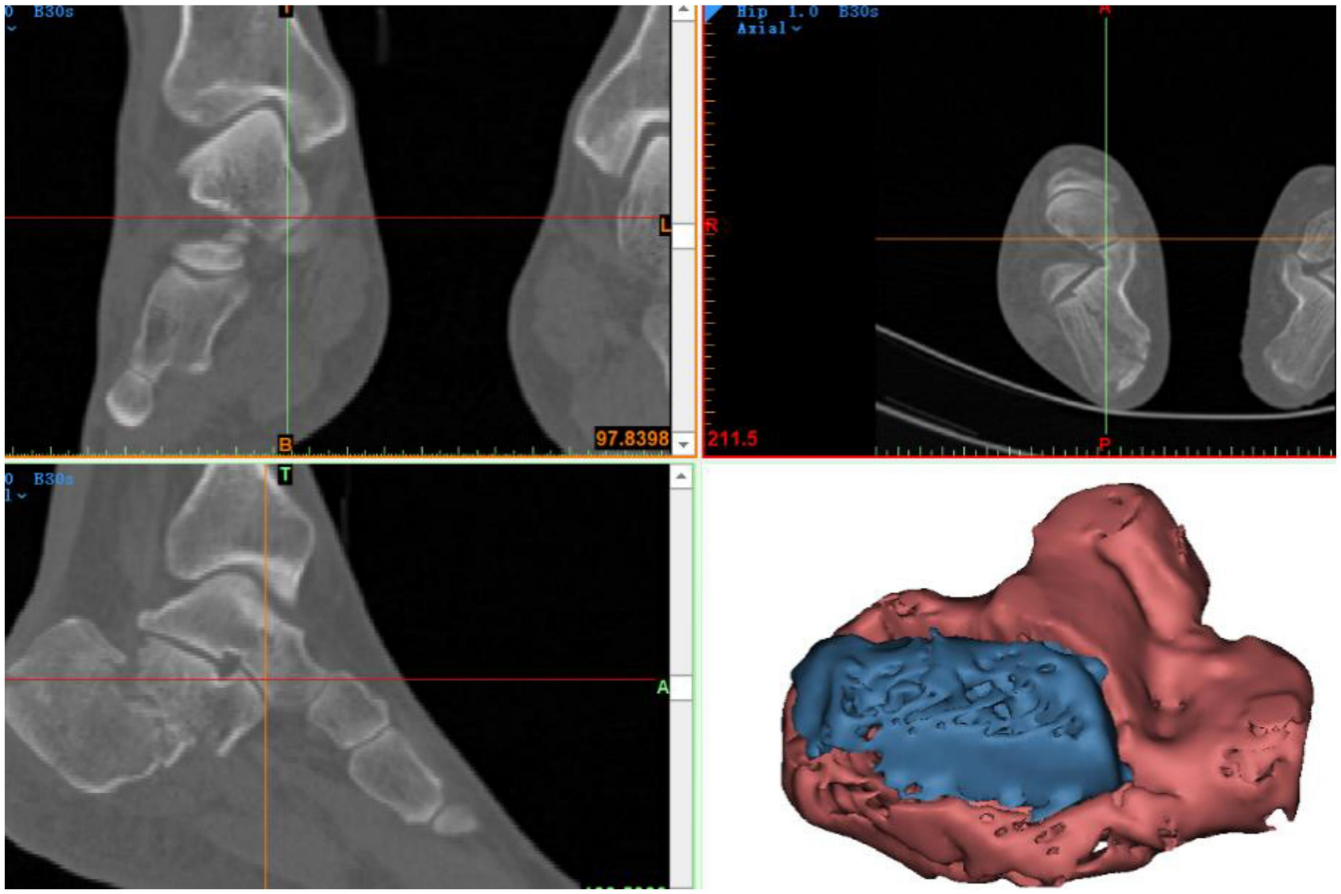
3D CT reconstruction.

Patient foot and ankle CT data (DICOM format) were imported into Mimics. An initial mask of the calcaneus was generated using the “Segment→CT Bone Segmentation” function based on CT Hounsfield unit (HU) thresholding. To refine the mask, manual editing tools (e.g., “Edit Mask”) were used to remove soft tissue artifacts and ensure precise bone segmentation. Subsequently, the posterior facet fragment was manually segmented from the calcaneal body along the fracture lines using the “Split Mask” function, guided by fracture line anatomy and imaging appearance. The operator iteratively verified the mask's accuracy to prevent model distortion from segmentation errors. Finally, the “Calculate Part” function converted the segmented masks into high-fidelity 3D models of the fragment and the intact calcaneus. A model resolution of 0.1 mm was used to meet the precision requirements of orthopedic clinical research. The segmented fracture models were extracted from CT data and exported as STL files for further analysis (Figure 1).

#### 2.2.2 Virtual anatomical reduction protocol

Virtual anatomical reduction of the posterior facet fragment was performed in 3-matic 13.0 using the “Interactive Translate” and “Interactive Rotate” functions. No external population-based anatomical template or single standard calcaneal model was used. Instead, the virtually reduced position was defined according to predefined anatomical reduction criteria, including restoration of posterior facet congruity, alignment with the sustentacular fragment and adjacent subtalar articular surface, restoration of calcaneal morphology, and recovery of conventional radiographic parameters. A reduction was considered acceptable when the following criteria were simultaneously satisfied on multiplanar and three-dimensional views: (1) restoration of posterior facet congruity with minimization of articular step-off and gap; (2) alignment of the posterior facet fragment with the sustentacular fragment and adjacent subtalar articular surface; (3) restoration of calcaneal height, width, and long-axis alignment; (4) correction of lateral wall expansion and gross cortical overlap; and (5) recovery of Böhler's and Gissane's angles toward their anatomical ranges.

The virtual reductions were performed by a senior orthopedic surgeon with more than 20 years of experience in foot and ankle trauma surgery. To assess interobserver reproducibility, a second independent orthopedic surgeon repeated the virtual reduction and measurement procedures in a randomly selected subset of 30 cases while blinded to the first observer's results.

#### 2.2.3 Definition and application of landmarks

**Figure 2:**
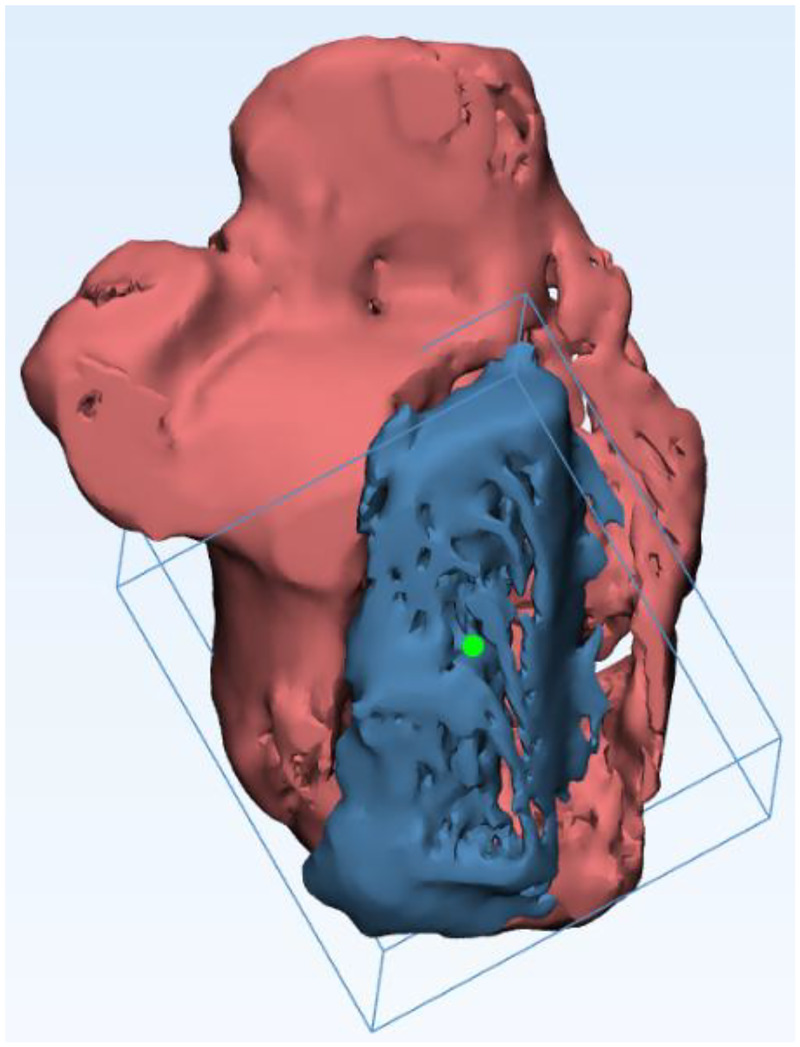
Landmark.

To quantify the spatial displacement, the geometric center of the posterior facet fragment was defined as a landmark using the “Create point” function, both before and after virtual reduction. This landmark selection, based on fragment morphology, ensured representative and reproducible measurements. The 3D coordinates (x, y, z) of the landmark were recorded to calculate the fragment's translational and rotational displacement (Figure 2).

#### 2.2.4 Standardization of model spatial position

**Figure 3:**
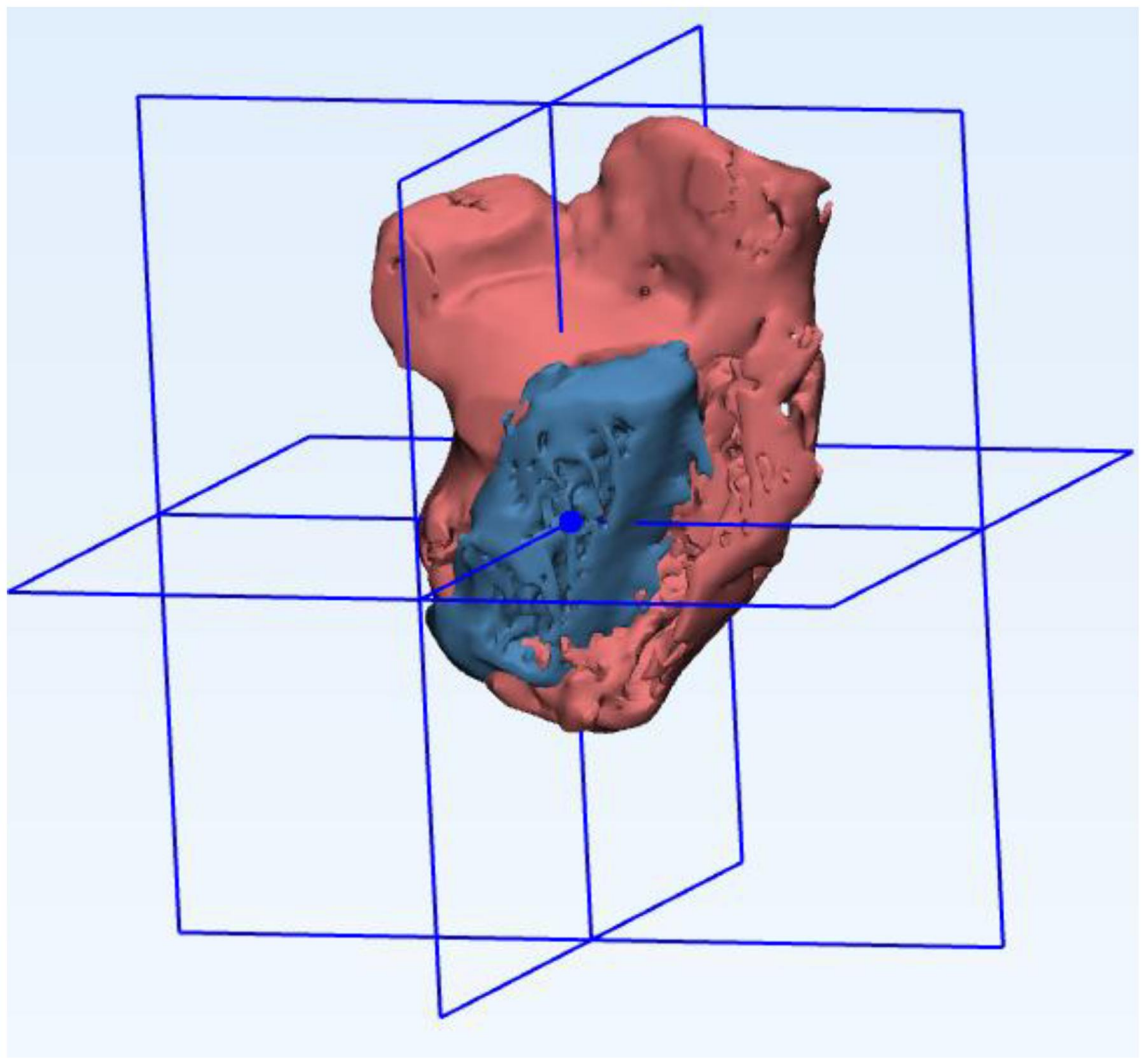
Standardized spatial position model.

To ensure the accuracy and consistency of subsequent measurements, the “Interactive Alignment” function was used to align the fracture model to a standard anatomical (standing) position. Reference planes (axial, sagittal, coronal) were then established using the “Create datum plane” function (Figure 3).

#### 2.2.5 Coordinate system and axis definitions

After reconstruction, all calcaneal models were standardized to an anatomical position. A right-handed coordinate system was established using the anatomical planes of the calcaneus. The mediolateral axis represented lateral-medial displacement, the anteroposterior axis represented posterior-anterior displacement, and the superoinferior axis represented inferior-superior displacement. Positive translational directions were defined as lateral, posterior, and inferior. Rotational displacement was calculated from the transformation matrix required to move the fragment from its fractured position to its anatomically reduced position. Rotation around the superior-inferior axis was defined as axial-plane internal or external rotation; rotation around the mediolateral axis was defined as sagittal-plane anterior or posterior tilt; and rotation around the anteroposterior axis was defined as coronal-plane varus or valgus.

To avoid directional inconsistency caused by side differences, left-sided fractures were mirrored to a right-sided coordinate system before rotational analysis. Therefore, the definitions of internal rotation, anterior tilt, and varus/valgus were consistent across all cases.

#### 2.2.6 Measurement of displacement distance and rotation angle

**Figure 4:**
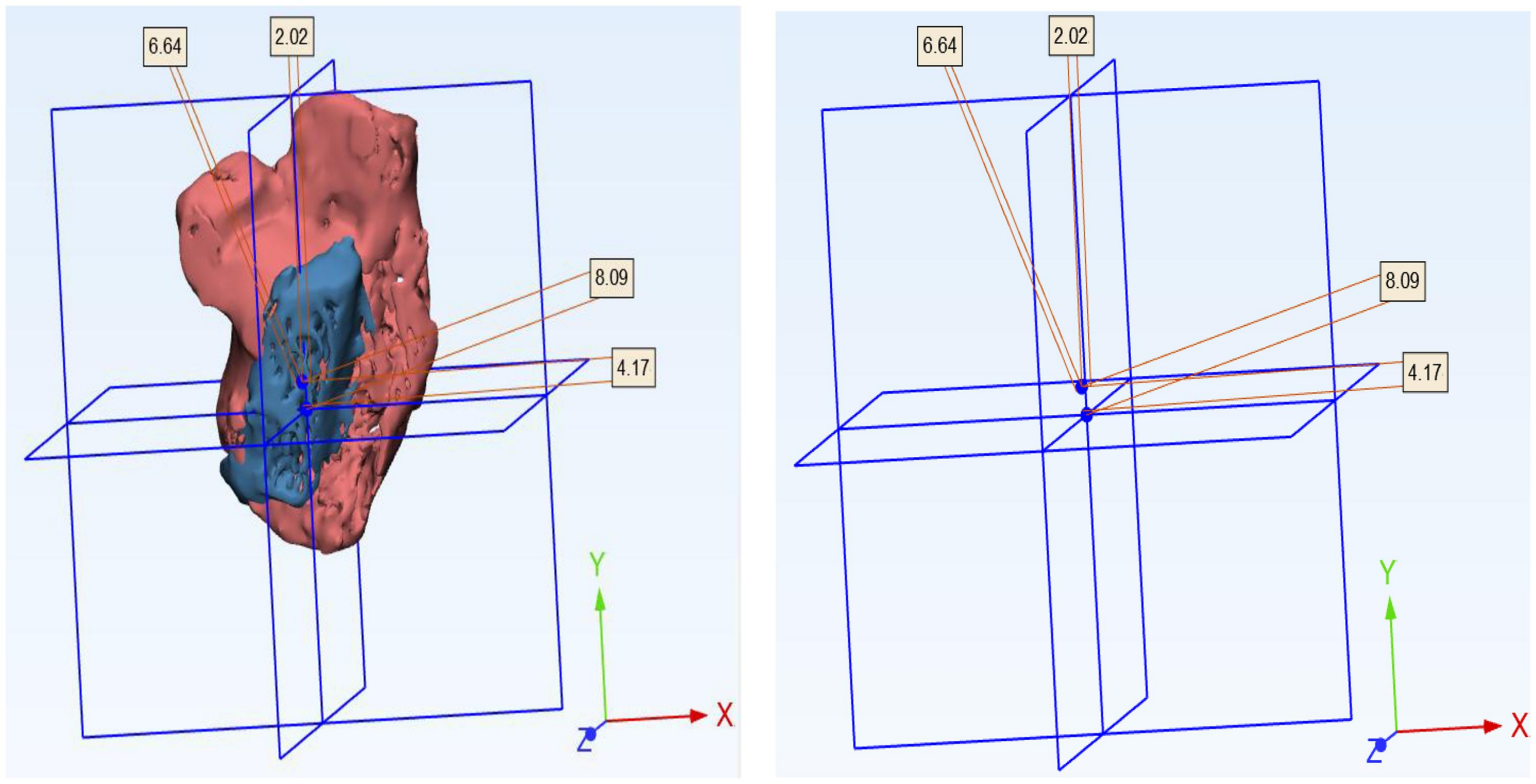
Schematic diagram of displacement distance measurement.

**Figure 5:**
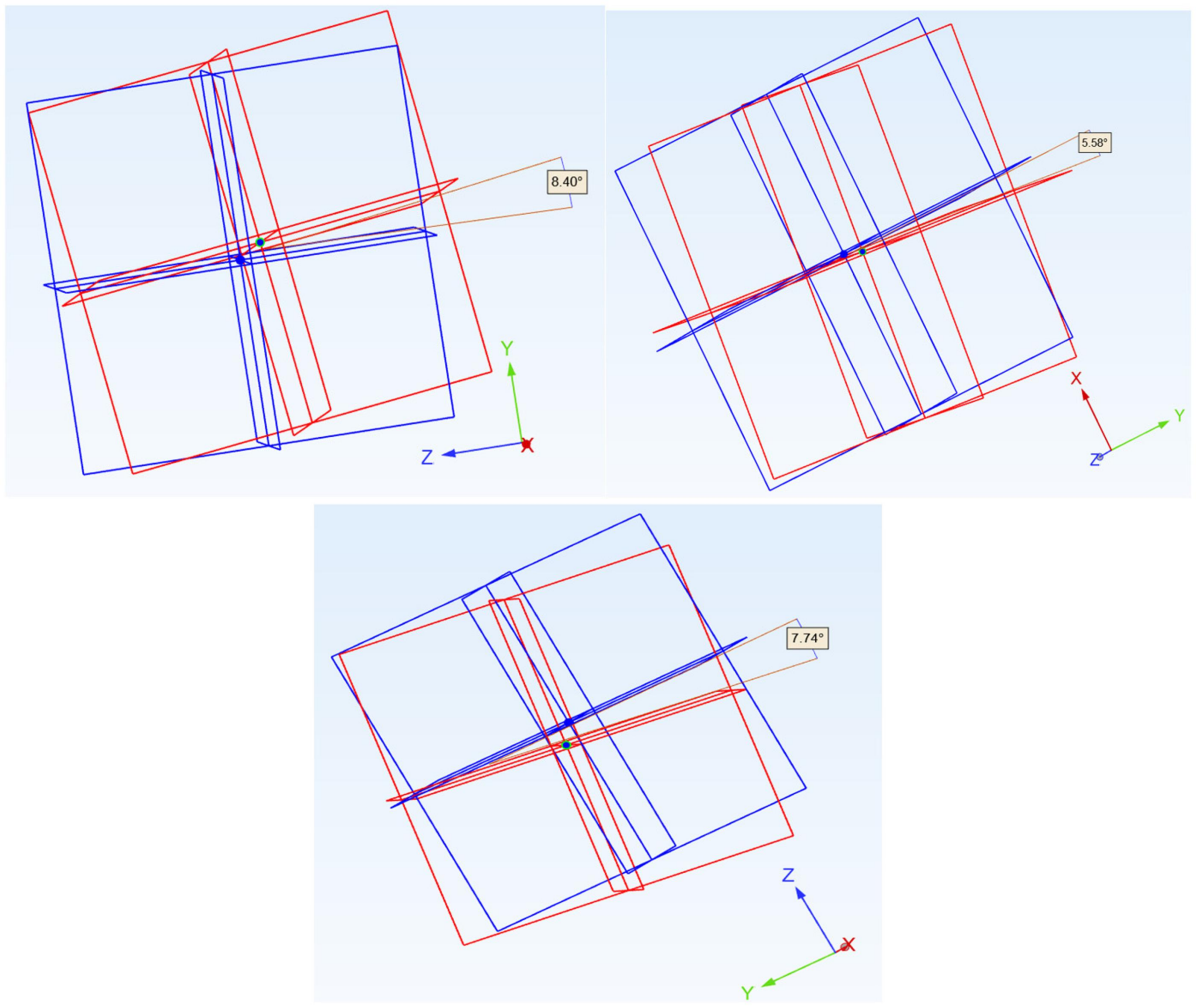
Schematic diagram of rotation angle measurement. The rotational component of the posterior facet fragment was quantified relative to its anatomically reduced position in the axial, sagittal, and coronal planes.

Based on the 3D coordinates of the landmarks and the standardized anatomical planes, we measured the overall displacement, as well as translations and rotations in the axial, sagittal, and coronal planes. Overall displacement was defined as the Euclidean distance between the landmark's pre- and post-reduction positions. Planar-specific translations were measured by projecting the landmark's coordinates onto each anatomical plane. To evaluate rotational displacement, rotation was defined as the angle of the fragment relative to its anatomically reduced position in all three planes. This was calculated as follows: the geometric center landmark was defined on the posterior facet fragment; virtual anatomical reduction was performed to align the fragment, restoring articular congruity and critical parameters such as Böhler's and Gissane's angles. The 3D coordinates of the landmark before and after reduction were recorded, and the rotational angles in the three planes were computed via a rotation matrix calculation. All displacement data were recorded in millimeters (mm) and rotational data in degrees (°), with a precision of 0.01 mm and 0.01°, respectively (Figures 4, 5).

#### 2.2.7 Reliability analysis

A random subsample of 30 cases was selected to evaluate interobserver reproducibility. The virtual reduction and measurement procedures were independently performed by two orthopedic surgeons who were blinded to each other's results. Interobserver reliability was assessed using the intraclass correlation coefficient (ICC), based on a two-way random-effects model with absolute agreement. Intraobserver reliability was not assessed in the present study.

### 2.3 Statistical analysis

Statistical analysis was performed using SPSS 26.0 (IBM Corp., Armonk, NY). Normally distributed continuous data were expressed as mean ± standard deviation (SD), while non-normally distributed data were expressed as median and interquartile range (IQR; P25, P75). Categorical data were presented as counts and percentages (%). One-sample t-tests (for normally distributed data) or Wilcoxon signed-rank tests (for non-normally distributed data) were used to evaluate whether fragment displacement and rotation significantly deviated from zero. A *P*-value < 0.05 was considered statistically significant. All statistical results were verified by a professional statistician.

Sanders classification was treated as a descriptive baseline variable. Because only 8 patients had Sanders type III fractures and the cohort was markedly imbalanced, no formal inferential subgroup comparison between Sanders type II and type III fractures was performed. Such an analysis would have been substantially underpowered and potentially misleading.

## 3 Results

### 3.1 Demographic characteristics

A total of 71 patients with calcaneal fractures were included. The mean age was 46.3 ± 10.4 years. The cohort was predominantly male (*n* = 66, 93.0%) compared to female (*n* = 5, 7.0%). Fractures were classified as Sanders type II (*n* = 63, 88.7%) or type III (*n* = 8, 11.3%). The injury involved the left side in 32 cases (45.1%) and the right side in 39 cases (54.9%). The majority of patients (*n* = 68, 95.8%) underwent surgical treatment, while 3 (4.2%) were treated non-operatively. Because the Sanders type III subgroup was small and the distribution between Sanders type II and type III fractures was highly imbalanced, Sanders classification was treated descriptively, and no formal subgroup comparison was performed. The baseline demographic and clinical characteristics of the 71 patients are summarized in Table 1.

**Table 1:** Baseline characteristics of 71 patients (*n*, %).

Basic Characteristics	Category	*n* (*n* = 71)	Percentage (%)
Sex	Male	66	93.0
	Female	5	7.0
Sanders classification	Type II	63	88.7
	Type III	8	11.3
Treatment	Non-operative	3	4.2
	Operative	68	95.8
Injured side	Left	32	45.1
	Right	39	54.9

### 3.2 Reliability of measurements

Analysis of the 30-case random subsample demonstrated good-to-excellent interobserver reliability for the translational and rotational measurements, with ICC values ranging from 0.806 to 0.871. These findings indicate that the three-dimensional virtual reduction and measurement workflow was reasonably reproducible between independent observers. Intraobserver reproducibility was not evaluated in the present study and is acknowledged as a methodological limitation.

### 3.3 Displacement characteristics of the posterior facet fragment

The displacement of the posterior facet fragment exhibited distinct patterns. The median overall displacement was 7.02 (IQR: 5.30, 8.71) mm. All planar translations were statistically significant (all *P* < 0.001), with lateral (4.55 ± 3.16 mm) and inferior (3.81 ± 3.13 mm) translations being predominant. Posterior translation (1.99 ± 2.83 mm) was less pronounced (Table 2).

**Table 2:** Displacement of the calcaneal fragment landmark.

Displacement direction	*n*	Displacement (mm)	t/Z	*P*
Lateral translation	71	4.55 ± 3.16	12.125	< 0.001
Posterior translation	71	1.99 ± 2.83	5.930	< 0.001
Inferior translation	71	3.81 ± 3.13	10.270	< 0.001
Overall displacement	71	7.02 (5.30,8.71)	7.323	< 0.001*

*Data are non-normally distributed; the Wilcoxon test was used.

Regarding rotation, all angular deformities were statistically significant (*P* ≤ 0.001). Axial plane internal rotation (17.62 ± 14.57°) and sagittal plane anterior tilt (12.29 ± 13.42°) were the most significant. Coronal plane varus (4.61 ± 13.97°) was minimal by comparison, but this measurement was associated with a high standard deviation and coefficient of variation, indicating significant instability in this plane. These results demonstrate that the posterior facet fragment displaces primarily in a lateral-inferior direction, with concomitant internal rotation and anterior tilt (Table 3).

**Table 3:** Rotation of the calcaneal fragment landmark.

Rotation direction	*n*	Rotation angle (°)	t	*P*
Axial plane internal rotation	71	17.62 ± 14.57	10.189	< 0.001
Sagittal plane anterior tilt	71	12.29 ± 13.42	7.712	< 0.001
Coronal plane varus	71	4.61 ± 13.97	2.781	< 0.001

## 4 Discussion

Current surgical treatments for IACFs include open reduction and internal fixation (ORIF) via an extended lateral approach, minimally invasive approaches with plate fixation, and percutaneous reduction and fixation techniques (16, 17). The traditional extended lateral approach allows for direct visualization and reliable reduction of the articular surface, facilitating stable fixation and rapid recovery (18). However, it requires extensive soft-tissue dissection and is associated with a high rate of postoperative complications (19, 20). Furthermore, the tenuous soft-tissue envelope and poor elasticity surrounding the calcaneus make the management of IACFs exceptionally challenging (21). Consequently, minimally invasive techniques have become a major focus of investigation (18, 22). These techniques necessitate percutaneous reduction of the displaced posterior facet fragment (16, 21).

Recent clinical studies have also emphasized the importance of restoring calcaneal morphology in multiple planes. Basal et al. reported mid- to long-term outcomes of a delta-frame triplanar external fixation technique for displaced intra-articular calcaneal fractures, particularly in Sanders type III and IV injuries with soft-tissue compromise. Their study demonstrated that triplanar restoration of calcaneal anatomy could achieve favorable radiological correction and functional outcomes. Although their work focused on clinical outcomes and external fixation rather than quantitative posterior facet fragment displacement, it provides relevant clinical context supporting the concept that calcaneal fracture reduction should be evaluated and corrected in three dimensions rather than in a single radiographic plane (23).

Posterior facet fractures are challenging orthopedic injuries. Fracture lines often propagate from the posterior facet into the calcaneal body, creating complex 3D displacements. The bulging and rotation of the lateral fragment are typical features, and their biomechanical consequences directly impact subtalar joint congruity (13, 24). Accurate assessment of these displacement characteristics is essential for surgical planning and restoring anatomical alignment; a precise understanding of the spatial displacement pattern is a prerequisite for achieving anatomical reduction. However, even for experienced foot and ankle surgeons, precise reduction of the posterior facet remains technically demanding, stemming from the limitations of conventional imaging and the complexity of the displacement. Traditional 2D CT assessment has clear drawbacks: displacement and rotation patterns are complex and superimposed, and 2D imaging can mislead surgeons into perceiving only simple, single-plane deformities. For example, a complex rotational deformity may be misdiagnosed as simple coronal varus. 3D-assisted surgery has been shown to improve the quality of reduction and stability of internal fixation, leading to better maintenance of reduction (25). Our study, through 3D quantitative analysis (Mimics+3-matic), systematically elucidates the spatial displacement patterns of the posterior facet fragment, identifying predominant displacement vectors and unstable rotational planes. This provides an evidence-based reference for precise intraoperative reduction.

Our translational data show that lateral (4.55 ± 3.16 mm) and inferior (3.81 ± 3.13 mm) displacements significantly exceeded posterior displacement (1.99 ± 2.83 mm). This finding is highly consistent with the biomechanics of axial loading injuries (13). It is crucial to distinguish the “Fall from Height” mechanism from Motor Vehicle Accidents (MVAs). Falls from height are generally dominated by axial loading, although the exact loading direction may vary according to landing posture, foot position, and surface condition. In contrast, motor vehicle accidents may involve additional dorsiflexion moments and anterior-posterior shear forces, which could result in different displacement vectors. Therefore, by restricting the cohort to falls from height, the present study reduced heterogeneity in injury mechanism; however, the findings should be extrapolated to motor vehicle accident-related fractures with caution. During a fall from height, axial load transmitted through the talus impacts the posterior facet, causing comminution and lateral wall blowout (26). Concomitant shear forces induce fragment translation in the transverse plane. Ringleb found a significant increase in subtalar translation (7.3 mm, *p* < 0.008) after transecting the anterior talofibular ligament (ATFL) and interosseous talocalcaneal ligament (ITCL), which aligns with our findings (27). Intraoperatively, rupture of the calcaneofibular ligament (CFL), a critical stabilizer of the lateral calcaneus, is often observed. Furthermore, the minimal muscle coverage of the calcaneus offers little resistance to lateral fragment displacement (28–30). This culminates in a composite injury pattern dominated by lateral and inferior translation (31). Notably, the median total displacement of 7.02 mm (IQR 5.30–8.71) far exceeds the 2 mm threshold associated with an increased risk of post-traumatic arthritis, highlighting that failure to reduce this displacement can lead to severe functional impairment and chronic pain (18, 32).

Our rotational data showed that axial-plane internal rotation (17.62 ± 14.57°) and sagittal-plane anterior tilt (12.29 ± 13.42°) were the predominant rotational components, whereas coronal-plane varus (4.61 ± 13.97°) was less pronounced but highly variable. The observed pattern may be partly explained by the oblique anatomical orientation of the posterior facet and the direction of axial loading during falls from height (32–34). Axial impaction transmitted through the talus may promote inferolateral translation of the posterior facet fragment and may be accompanied by a coupled rotational component (32, 33). In addition, previous studies on subtalar joint stability and foot-ankle biomechanics suggest that ligamentous constraints and tendon-related forces may influence hindfoot motion under load (27, 29, 35, 36). However, these factors should be regarded only as possible biomechanical explanations rather than direct mechanistic conclusions, because the present study did not include cadaveric loading experiments, finite element analysis, or direct assessment of ligamentous and tendinous injuries. Therefore, the proposed helical displacement pattern requires further biomechanical validation.

Our mean varus angle (4.61 ± 13.97°) differs substantially from the 21.9° reported by Shi (37). We attribute this discrepancy to their small sample size (*n* = 6) and the inherent variability of coronal plane rotation. Indeed, the high standard deviation (13.97°) for coronal rotation in our cohort resulted in a coefficient of variation (CV = 303%)—far exceeding those of the other two planes—suggesting significant instability.

The high variability of coronal-plane rotation may reflect heterogeneity in fracture morphology, fragment size, comminution pattern, and the subjectivity inherent to virtual reduction. It may also explain why coronal-plane deformity is more difficult to generalize than axial-plane internal rotation or sagittal-plane anterior tilt. Further biomechanical and clinical studies are required to clarify the factors contributing to varus-valgus variability (29, 38). Concurrently, Ringleb et al. observed that CFL transection significantly increased varus displacement (4.53 mm, *p* < 0.027), whereas CFL tension could conversely increase valgus (27).

The sagittal-plane anterior tilt quantified in this study may provide a three-dimensional anatomical explanation for the reduction in Böhler's angle and the increase in Gissane's angle observed on conventional imaging. Therefore, restoring Böhler's angle (normal: 30–40°) and Gissane's angle (normal: 120°–130°) remains a core objective of fracture reduction. Posterior facet collapse and anterior tilt directly cause a significant decrease in Böhler's angle and an increase in Gissane's angle (39, 40). A Böhler's angle ≤ 21° demonstrates 99% sensitivity and specificity for fracture diagnosis (41). The degree of preoperative Böhler's angle reduction correlates positively with Sanders classification severity, and a severely reduced initial angle is associated with poorer 2-year functional outcomes regardless of treatment (39, 42). Surgical standards require simultaneous restoration of both angles (43–45). This involves prioritizing anatomical reduction of the posterior facet (using the talar facet as a reference), restoring Gissane's angle by reducing the subtalar joint collapse, applying lateral compression, and using leverage (e.g., prying) to correct the anteriorly tilted fragment, thereby restoring the calcaneus's 3D morphology (40, 46). Bone grafting may be used if necessary to maintain angular stability (43). Changes in these angles directly reflect the severity of articular collapse and fragment tilt. A postoperative Böhler's angle ≥30° is a decisive factor for functional prognosis and must be achieved through precise anatomical reduction and mechanical stability (43, 45, 47).

From a clinical perspective, the present findings suggest that posterior facet reduction may require attention not only to vertical depression and lateral translation but also to rotational malalignment, particularly axial-plane internal rotation and sagittal-plane anterior tilt. These quantitative data may provide an anatomical reference for preoperative planning and intraoperative assessment during minimally invasive reduction. However, because this study did not evaluate postoperative reduction quality, subtalar congruity, functional outcomes, or long-term prognosis, no definitive conclusion can be drawn regarding whether a specific derotation maneuver improves clinical outcomes. Prospective clinical studies are needed to clarify the relationship between correction of rotational deformity and patient outcomes.

It should be noted that the present cohort was predominantly composed of Sanders type II fractures, whereas Sanders type III fractures accounted for only a small proportion of cases. Increased comminution in Sanders type III fractures may theoretically influence both translational displacement and rotational instability of the posterior facet fragment. However, because only 8 Sanders type III fractures were included, a statistically meaningful subgroup comparison could not be performed. Therefore, the displacement pattern identified in this study should be interpreted primarily as reflecting a Sanders type II-dominant cohort, and its applicability to more comminuted Sanders type III fractures should be considered cautious and hypothesis-generating.

This study possesses several limitations. First, the retrospective design relied on medical records to classify injury mechanism and did not include detailed biomechanical variables such as fall height, landing posture, or landing surface properties. Second, the cohort showed a marked imbalance in Sanders classification, with a predominance of Sanders type II fractures and only 8 Sanders type III fractures. Therefore, no reliable subgroup analysis according to Sanders classification could be performed, and the findings should be interpreted primarily as reflecting a Sanders type II-dominant cohort. Third, because the reduced model was generated through virtual anatomical reduction, the measured translational and rotational displacement may partly depend on the predefined reduction criteria and observer judgment. Although predefined anatomical criteria and interobserver reliability analysis were used to reduce this potential bias, future studies using contralateral mirror-image models, cadaveric validation, or intraoperative/postoperative CT confirmation would further improve measurement validity. Fourth, intraobserver reproducibility was not assessed, which limits the ability to fully evaluate the repeatability of the virtual reduction and measurement workflow. Finally, the present study did not evaluate postoperative reduction quality, subtalar congruity, functional outcomes, or long-term prognosis; therefore, the clinical benefit of targeted correction of rotational deformity remains to be determined.

## 5 Conclusion

In this Sanders type II-dominant cohort of intra-articular calcaneal fractures caused by falls from height, the posterior facet fragment demonstrated a three-dimensional displacement pattern characterized mainly by lateral and inferior translation, together with axial-plane internal rotation and sagittal-plane anterior tilt. These findings suggest that rotational malalignment may be an important but easily overlooked component of posterior facet displacement. The present quantitative data may provide an anatomical reference for preoperative planning and intraoperative reduction assessment, particularly in minimally invasive procedures. However, because Sanders type III fractures were underrepresented and no postoperative clinical outcomes were evaluated, the applicability and clinical benefit of targeted rotational correction require further validation in future biomechanical and prospective clinical studies.

## Data Availability

The raw data supporting the conclusions of this article will be made available by the authors, without undue reservation.
